# Silver/Chitosan Nanocomposites: Preparation and Characterization and Their Fungicidal Activity against Dairy Cattle Toxicosis *Penicillium expansum*

**DOI:** 10.3390/jof6020051

**Published:** 2020-04-19

**Authors:** Mousa A. Alghuthaymi, Kamel A. Abd-Elsalam, Ashwag Shami, Ernest Said-Galive, Eleonora V. Shtykova, Alexander V. Naumkin

**Affiliations:** 1 Biology Department, Science and Humanities College, Shaqra University, Alquwayiyah 11726, Saudi Arabia; malghuthaymi@su.edu.sa; 2 Plant Pathology Research Institute, Agricultural Research Center (ARC), Giza 12619, Egypt; 3 Biology Department, College of Sciences, Princess Nourah bint Abdulrahman University, Riyadh 11617, Saudi Arabia; 4 A.N. Nesmeyanov Institute of Organoelement compounds (INEOS) of Russian Academy of Sciences, 119991 Moscow, Russia; ernest@ineos.ac.ru (E.S.-G.); naumkin@ineos.ac.ru (A.V.N.); 5 V. Shubnikov Institute of Crystallography of Federal Scientific Research Centre “Crystallography and Photonics” of Russian Academy of Sciences, 119333 Moscow, Russia; shtykova@ns.crys.ras.ru

**Keywords:** citrinin, nanocomposites, mycotoxins, patulin, *P. expansum*

## Abstract

This work aimed to evaluate the fungicide activity of chitosan-silver nanocomposites (Ag-Chit-NCs) against *Penicillium expansum* from feed samples. The physicochemical properties of nanocomposites were characterized by X-ray fluorescence analysis (XRF), small-angle X-ray scattering (SAXS), X-ray photoelectron spectroscopy (XPS), and transmission electron microscopy (TEM). The morphological integrity of the nanohybrid was confirmed by electron transmission. By the data of RFA (X-ray fluorescent analysis), the contents of Ag in Ag-chitosan composite were 5.9 w/w%. The size distribution of the Ag nanoparticles incorporated in the chitosan matrix was investigated by SAXS. The main part of the size heterogeneity distribution in the chitosan matrix corresponds to the portion of small particles (3–4 nm). TEM analysis revealed a spherical morphology in the form of non-agglomerated caps, and 72% of the nanoparticles measured up to 4 nm. The minimum inhibitory concentration of NCs was evaluated in petri dishes. Three different concentrations were tested for antifungal activity against the mycotoxigenic *P. expansum* strain. Changes in the mycelium structure of *P. expansum* fungi by scanning electron microscopy (SEM) were observed to obtain information about the mode of action of Ag-Chit-NCs. It was shown that NC-Chit-NCs with sizes in the range from 4 to 10 nm have internalized sizes in cells, form agglomerates in the cytoplasm, and bind to cell organelles. Besides, their ability to influence protein and DNA fragmentation was examined in *P. expansum*. SDS-PAGE explains the apparent cellular protein response to the presence of various Ag-Chit-NCs. The intensity of *P. expansum* hyphal cell protein lines treated with Ag-Chit-NCs was very thin, indicating that high molecular weight proteins are largely prevented from entering the electrophoretic gel, which reflects cellular protein modification and possible damage caused by the binding of several protein fragments to Ag-Chit-NCs. The current results indicate that Ag-Chit-NCs <10 nm in size have significant antifungal activity against *P. expansum*, the causative agent of blue mold-contaminated dairy cattle feed.

## 1. Introduction

Post-harvest fungal pathogens, such as Penicillium expansum (Link) Thom., are one of the most well-known microbes in the ecosystem and one of the first fungal species identified in the Penicillium genus [1]. *P. expansum* is found to manifest in various types of food and taken and adopted in different climate zones [2]. Mycotoxins, which are produced by various Penicillium species, such as *P. roqueforti*, *P. paneum*, and *P. expansum*, have been reported in silage and have caused animal health problems depending on the year of sampling and forage species [3]. Two types of fungi can produce roxfortin C (ROC), and *P. roqueforti* also produce PR toxin and mycophenolic acid (MPA), whereas *P. paneum* and *P. expansum* produce patulin [2,3]. *P. expansum* usually occurs in soil and is associated with some moldy fruits and vegetables, especially rotten apples and figs. It is the main source of citrinin and patulin in natural products [4]. *P. expansum* has also been isolated in fish feed, and the use of vegetable waste as a nutrient may be the reason for the existence of this species in processed fish feed [5]. Food and feed contamination with patulin and citrinin is quite low, but patulin occurs in apple juice, apples, and pears that are exposed to brown rot, as well as in grapes, flowers, and animal feed during storage [4]. Mycotoxins, which are produced by certain Pencillium species, for example, patulin, PR toxin, roxfortin, and mycophenol, are known for their high ability to cause mycotoxicosis in animals. Synthetic chemical antimicrobials are broadly utilized in conventional agriculture to manage plant diseases. However, environmental dangers caused by the immoderate use of chemical antimicrobial agents pose health problems as the current society is becoming more health conscious. Therefore, agricultural researchers are developing green and eco-friendly techniques to control plant diseases [6]. Nanomaterials have received a unique interest because of their exciting physical and chemical properties. Nanotechnology has benefited the area of food protection due to the construction of new antifungal agents, particularly for the reduction of chemicals fungicides, and similarly, it has been used to absorb mycotoxins [7,8]. Colloid solutions from silver nanoparticles (Ag NPs) containing concentrations up to 35 ppm have been found to have inhibitory effects on three fungal genera, including *Aspergillus*, *Penicillium*, and *Trichoderma* [9]. The minimum growth inhibition concentration of silver nanocomposite, silver nitrate, and silver zeolite for *Aspergillus niger* was determined by Egger et al. (2009) [10]. Ag NPs have been shown to have strong antifungal properties. The effects of Ag NPs on plant pathogenic spores of *Fusarium culmorum* have been described by Kasprowicz et al. (2010) [11,12]. They are safe inorganic antimicrobials and can kill more than 650 species of microorganisms [13]. Ag NPs are one of the most systematically studied nanomaterials and have gained popularity because of their biocidal activity against fungi [14,15]. Chitosan silver nanoparticles have been examined for the control of fungal pathogens that were transmitted from seeds to beans. Significant antifungal activity against *Aspergillus flavus*, *R. solani*, and *Alternaria alterneta* was alternatively observed [16]. The antifungal properties of silver-chitosan nanoparticles functioned with 4(E)-2-(3-hydroxynaphthalin-2-yl)diazen-1-yl) and benzoic acid were evaluated for the control of *Aspergillus flavus* and *A. terreus*. An inhibition of fungal growth was shown between 20.2 and 27.0 mm [17]. The chitosan antifungal activity in combination with silver to combat *Colletotrichum gloeosporioides* was evaluated using mangoes (*Mangifera indica*). The two concentrations showed a reduction in anthracnose symptoms by 45.7 % and 71.3 %, respectively [18]. The antifungal activity of silver ions against two strains of pathogenic fungi, namely *Aspergillus avus* and *Penicillium vulpinum*, and the effectiveness of silver ions in inhibiting the production of AFB1 and patulin by two toxigenic fungal strains was tested in vitro. Additionally, silver ions have antifungal activity in the production of AFB1 and patulin [19]. There has been no past report on the utilization of Ag-Chit-NCs against toxic strains from *Penicillium* ascomycetous fungi. Ag NPs are extremely powerful against phytopathogens with low poisonous quality and lead to a broad range of applicability in pesticidal activity. It is proficiently utilized for site-targeted delivery of significant agrochemical substances [6,18]. Likewise, silver NPs embedded in a polymer network (Ag NP/polymer nanocomposites) are brilliant competitors as antimicrobial substances and have been used in food packaging to improve the timeframe of realistic usability of food, and the management toxigenic fungi and plant pathogens [6,7,20,21]. The main aim of the current research was to synthesize and characterize chitosan-silver nanocomposite using a supercritical carbon dioxide medium. The physicochemical properties of the produced nanocomposites were characterized by X-ray fluorescence analysis (XRF), small-angle X-ray scattering (SAXS), X-ray photoelectron spectroscopy (XPS), and transmission electron microscopy (TEM). The fungicidal impact of Ag-Chit-NCs was assessed against *Penicillium expansum* strains. The protein and DNA degradations were measured utilizing acrylamide and agarose gel electrophoresis, respectively.

## 2. Materials and Methods

### 2.1. Synthesis and Characterization of Ag NPs-Chitosan Nanocomposites

High molar mass chitosan (HMWC) (“Acros Organics”), a powder fraction with a size of 40 μm and less with MM of 600–800 KDa and DD >75%, was used as a matrix. The organometalic complex (1,5-cyclooctadiene)(1,1,1,5,5,5-hexafluoroacetylacetonato)silver(I) CODAg(hfacac), a greyish crystalline compound, m.p. 122–124 °C, solubility in SC CO_2_ ~10^−5^ mol/L at 14 MPa, and 70 °C (according to our data), was used as a metal precursor for synthesis in SC CO_2_ medium. The complex was purchased from Aldrich and used without additional purification. Carbon dioxide with a purity of 99.997 vol. % and hydrogen of high purity (H_2_ volume fraction not less 99.999%) were purchased from Moscow Plant of pure gases “Linde gas”. Acetone was distilled before use. 

### 2.2. Experimental Procedure

The synthetic fluid technology for producing metal nanocomposites includes 4 stages: (1) chitosan drying in vacuum at the temperature of 373 K; (2) impregnation of chitosan with the complex CODAg(hfacac) in SC CO_2_ solution (353 K, 20 MPa, 7 h), decompression; (3) purge of the reactor with hydrogen flow and the complex reduction in H_2_ atmosphere(1.2 MPa at the temperature of 353 K, 7 h); and (4) washing with ethanol and acetone, and vacuum drying the Ag-chitosan NC at a temperature of 333 K. After the first stage, chitosan lost water (9 w/w%% by TGA data). The second stage was carried out with 4.24 g (0.026 M) of chitosan and 1.059 g (2.5 × 10^−3^ M) of a silver complex (25% from chitosan weight). The calculated ratio was one silver atom per 10.4 chitosan units. Chitosan, the silver complex, and stir bar were loaded into the reactor on its bottom. The reactor volume was 25 cm^3^. The impregnated polymer after the reduction stage was washed with ethanol and acetone to remove the residual unreduced complex and degradation products. Impregnation and reduction were carried out according to the procedure described elsewhere [22].

### 2.3. X-Ray Fluorescence Analysis (XRF)

Metal content was determined using XRF on a “VRA 30” X-ray fluorescent analyzer (Leipzig, Germany) from the line of the AgKα spectrum of X-ray fluorescence (XF). To excite XF, an X-ray tube with a Mo anode was used in the 50 kV, 20 μA regime. The powdered chitosan samples containing metal nanoparticles were analyzed as pressed pills. The reference sample for spectrometer calibration was made from a polystyrene and metal salt fine mix, containing *n*% of metal, with a layer of reference sample with a mass of 1.23 mg. The Ag Kα peak height was created by the metal with a mass of 1.23 × 0.*n* = mass of metal (mg). The mass (and thus the concentration) of metal in the experimental sample was determined by comparing the peak height of the composite with that of the reference sample.

### 2.4. Small-Angle Spread (SAXS)

SAXS measurements were carried out with an AMUR-K diffractometer (designed by the Crystallography Institute of the Russian Academy of Sciences) at a wavelength of *λ* = 0.1542 nm with Kratky geometry (infinitely long slit). The heterogeneity of the size distribution was shown in the sample and their Cu nanoparticles were calculated using an indirect GNOM transformation program [23]. More detailed information about the experiments and calculations was explained earlier by Said-Galiev et al. (2011) [24].

### 2.5. X-Ray Photoelectron Spectroscopy (XPS)

The X-ray photoelectron spectrum was obtained with an Ultra DLD Axis spectrometer (Kratos, UK) using Al Kα (1486.6 eV) monochromathic radiation with an operating power of 150 W on the X-ray tube. High-resolution studies and corresponding to core spectrums were recorded at energies of 160 and 40 eV and with step sizes of 1 and 0.1 eV, respectively. The sample area of 300 μm × 700 μm contributed to the spectrum. The reference pressure in the analytical UHV chamber of the spectrometer during the measurement did not exceed 10^−8^ Torr. The spectrometer energy scale was calibrated to provide the following reference sample values (i.e., metal surfaces freshly cleaned by ion bombardment): Au 4f_7/2_–83.96 eV, Cu 2p_3/2_–932.62 eV, Ag 3d_5/2_–368.21 eV. Sample loading was improved by reference to the C-C/C-H peak developed in the C1s spectrum (284.8 eV). After cost abstraction, Shirley’s high-resolution background with inelastic losses was reduced from the high-resolution spectrum. The Cu LMM and Zn LMM Auger spectrum were corrected using a linear background. The chemical composition of the surface was calculated using the atomic sensitivity coefficient contained in the spectrometer software, which was corrected for the instrument transfer function of the instrument.

### 2.6. Transmission Electron Microscope (TEM)

Powdered samples were ground in an agate mortar and then placed on a film-coated 200-mesh copper specimen grid. TEM micrographs were performed on Carl Zeiss Leo 912 AB OMEGA electron microscope at an accelerating voltage of 80 kV. 

### 2.7. Pencillium Identification

Colony appearance, exudate formation, pigmentation, and re-staining were evaluated and the colony diameter measured and recorded after one week of growth at 25 °C. Five plates were inoculated at 25 °C, with the microscopic slides made from 7-day PDA culture. Conidia morphology, type of conidiophores, color, and phialid shapes were evaluated, and conidia were measured with a Leica microscope, which was taken with a Leica DC 300 digital camera and Leica IM 1000 software equipped. Morphological identification of each isolate was carried out according to the method described by Visagie et al. (2014) [25].

### 2.8. Patulin- and Citrinin-Producing Ability

Seventeen *Penicillium* isolates collected from feed samples were grown in SKMY liquid media (200 g sucrose, 0.5 g magnesium sulfate, 3 g potassium nitrate, 7 g yeast, and 1000 mL distilled water extract) for 10 days at 25 ± 2 °C After the incubation period, the contents of all flasks were blended on high speed for 2–3 min and mixed with 5 g of sodium chloride and then filtered on glass filter paper. Then, 100 mL of the fungus filtrate was centrifuged at 3500 g at 4 °C for 10 min. The top layer was removed, and the sample subsequently diluted 20 times (*v*/*v*) with deionized water. The suspension was filtered (Millipore, 0.45 μm) and the filtrate was centrifuged for 15 min at 15 °C at 2700× *g* and the top phase was removed and a water-methanol layer (100 μL) was added to 0.01 M phosphate-buffered saline (PBS) (900 μL; dilution 1:10). Patulin and citrinin were assayed in 100 μL of the prepared solution.

**Figure 1:**
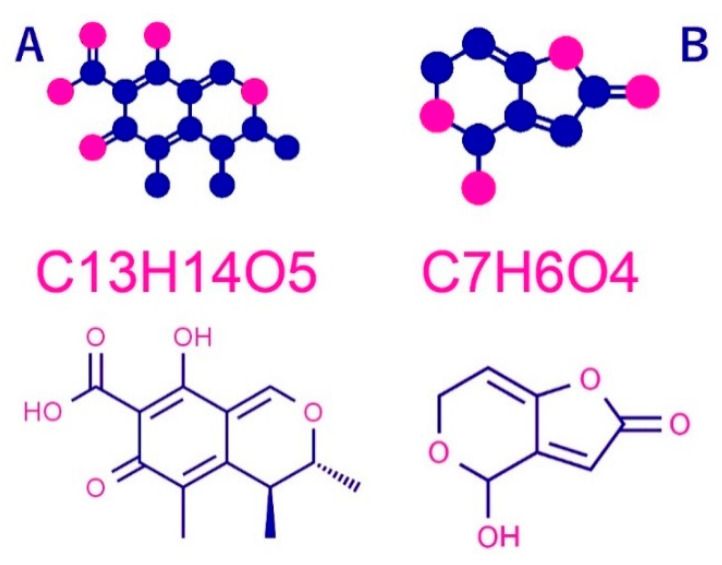
Structural formula of patulin and citrinin (**A**) Patulin, (2,4-dihydroxy-2H-pyran-3 (6H) ylidene) acetic acid, (**B**) Citrinin, acid 3,4-lactone, (3R, 4S)-7-(Dihydroxymethylene))-3,4,5-trimethyl-3H-isochromic-6,8 (4H, 7H)-dione. Red dot (H) Blue dot (C). Data available online from: http://www.chemspider.com.

Broth filtrate for the cultivation of the most commonly isolated *Pencillium* species, which was taken from various feed samples, was analyzed for poisons from patulin and citrinin (see the chemical formula in Figure 1. The mycotoxin productivity from 17 penicillium isolates representing 5 species was tested by HPLC (PerkinElmer^®^ Brownlee ™ (Waltham, MA 02451, USA) validated C18, 250 mm, equipped with a UV detector. The total separation time was about 25 min with a flow rate of 1 mL/min). The isolates were cultured aseptically in triplicate in a 100-mL flask of malt extract broth and incubated for 10 days at 27 ± 2 °C. The cultures were mixed for 2 min using high-speed homogenizers and filtered using glass filter paper. Patulin was extracted from homogenized filtrate using acetonitrile:water solution (5:95 *v*:*v*). The solvent was then evaporated in a vacuum at 35 °C. Residues containing dry patulin were dissolved in 1 mL of acetonitrile:water (5:95 *v*:*v*). The extract was then passed through a 0.45-μm microfilter before HPLC analysis. The method described by Christian (1990) [26] was used to determine patulin. Citrinin was extracted from the homogenized filtrate using dichloromethane with the addition of phosphoric acid and the extract was then purified in a polyamide column. HPLC analysis of citrinin employed the method described by Franco et al. (1996) [27].

### 2.9. Screening for Antifungal Activity

To investigate the antifungal effects of Ag-Chit-NCs on *P. expansum* isolates, three chitosan NCs suspension concentrations (0.30, 0.60, and 0.100 mg·mL^−1^) were prepared. The antifungal activity of Ag-Chit-NCs was detected by calculating the decrease in fungal growth of two pathogenic species using agar-well diffusion tests (Perez et al., 1990). A 5 mm diameter well was made in a Petri dish, 30 μm. Each concentration of Ag-Chit-NCs was added to the agar. The plates were inoculated with fungal discs from *P. expansum* isolates and incubated at 28 °C for 10 days. Positive control media (without chitosan) were inoculated in the same way. The rate of growth inhibition was measured and calculated using the following formula [28]:

Growth Inhibition (%) = (*C* − *P*)/*C* × 100, where *C* is the diameter of mat growth in the control plate and *P* is the diameter of micelle growth in the processed plate. Five treatment repetitions were used, and the present assay was repeated three times.

### 2.10. Protein Degradation Test

Protein damage in the fungal cells of *P. expansum* incubated with Ag-Chit-NCs was examined using SDS-PAGE, which was carried out according to the protocol published by Laemmli (1970) [29]. SDS-PAGE was performed using a 5–10% gradient of polyacrylamide gels containing 0.1% SDS. Proteins were analyzed in 1.5 mm and 15 cm gels that worked in double vertical plates (Hoefer Scientific Instruments, San Francisco, CA, USA). Then, 25 μL of protein extract from each sample was put into a polyacrylamide gel. Protein was electrophoresed at a constant electric current of 30 mA through stacking gel, and at 90 mA through separating gel at room temperature, and the gel was stained with silver staining [30]. The standard molecular weight used for gel analysis was the Sigma protein marker, which ranged between 66,000, 45,000, and 22,000 kDa.

### 2.11. Binding/Degradation of Genomic Fungal DNA

The fungal mats of *P. expansum* were prepared in 20 mL of liquid media (24 g·L^−1^ potato dextrose broth (PDB, Difco Laboratories)). Mycelium was harvested by filtration through mesh sieves (40 μm), washed with sterile water, and deposited onto Whatman paper to eliminate excess water. Mycelium was ground to a fine powder in liquid nitrogen using a mortar and pestle. Fungal DNA was extracted by the method of Moslem et al. (2010) [31]. To check the DNA quality, 10 μl of *P. expansum* DNA were treated with Ag-Chit-NCs (40 μg·mL^−1^) for 2 h at 37 °C. The products resulting from the interactions of the NPs with DNA were separated by 1.5% (*w*/*v*) agarose gel containing 0.05 μg·mL^−1^ ethidium bromide. Gel Documentation and Analysis Systems, Uvitec (Cambridge, UK) were used to capture the image.

### 2.12. Scanning Electron Microscopy (SEM) 

Penicillium biomass obtained from fungal cultures grown on PDB treated with and without Ag-Chit-NCs for one week was used for a scanning electron microscopy (SEM) test to demonstrate the probable modes of action. About 5 × 10 mm segments were cut from a culture grown on PDA plates and immediately inserted in bottles containing 3% glutaraldehyde in 0.05 M phosphate buffer (pH 6.8) at 4 °C. Samples were held in this solution for 48 h to be repaired and then washed three times for 20 min each with distilled water. Samples were then dehydrated for 20 min in the ethanol series dilution (30%, 50%, 70%, and 95%) at each dilution with ethanol and finally in absolute ethanol for 45 min. The sample was then critically dried in liquid carbon dioxide. Penicillium fragment segments were placed in a desiccator until further use. After drying, the prepared samples were installed using standard double-sided adhesive on standard ½-inch SEM nozzles and with gold-palladium galvanoplastics (60 s, 1.8 mA, 2.4 kV) in the coated Poutron SEM Coat coating system. All samples were examined in JEOL JXA-480 SEM (JEOL, Tokyo, Japan), which operated at 15 kV at 6000-fold magnification. SEM works in the SEM department of the National Research Center’s Central Unit for Scientific Analysis and Services in Giza, Egypt. JEOL JXA-480 SEM.

### 2.13. Statistical Analyses

The experimental design was a randomized complete block, with five replications in the greenhouse assay and five replications in the laboratory test. Data were subjected to analysis of variance (ANOVA). Least significant difference (LSD) was used to compare concentration means within genotypes. ANOVA was performed with MSTAT-C statistical package, Version 2.0.0.

## 3. Results

### 3.1. Physico-Chemical Characterization of Silver/Chitosan Nanocomposites

**Figure 2:**
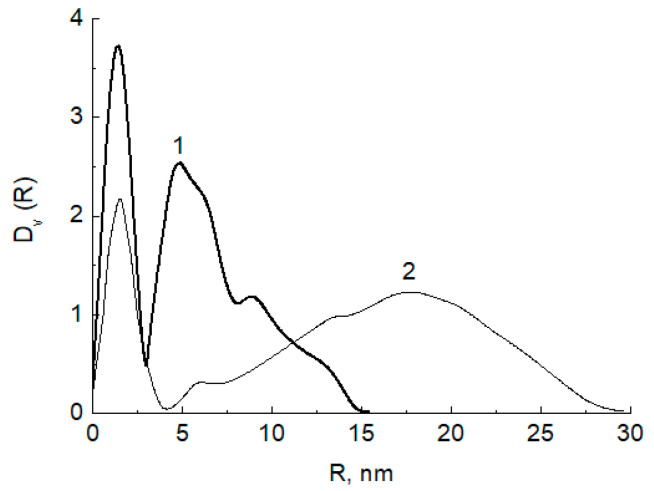
1—volume size distribution function of the scattering heterogeneities in initial high molecular weight chitosan after exposition in SC CO_2_ (25 MPa, 80 °C); 2—size distribution function D _v_ (R) of the high molecular weight chitosan sample after impregnation with Ag-complex and reduction with hydrogen (1.15 MPa, 65 °C). Sample 1: chitosan (HMWC), Sample 2: chitosan matrix.

From the data of RFA (X-ray fluorescent analysis), the contents of Ag in Ag-chitosan composite were 5.9 *w*.%. The size distribution of Ag nanoparticles incorporated in the chitosan matrix was investigated by SAXS. The volume size distribution functions of scattering heterogeneities in the initial high molecular mass chitosan after exposition in SC CO_2_ (1) and of the Ag nanoparticles impregnated with COD Ag[hfacac] in the chitosan matrix (2) were calculated from the scattering data using GNOM program. The results of the size distribution analysis are given in Figure 2

As shown in Figure 2, the volume size distributions of both scattering inhomogeneities in the initial chitosan matrix (Figure 2, curve 1) and Ag nanoparticles formed in it (Figure 2, curve 2) demonstrate a bimodal character: Two fractions of small and large scattering inhomogeneities were observed (Table 1). However, the initial chitosan matrix is characterized by a much wider size distribution containing inhomogeneities with sizes up to 60 nm. Interesting, after the reduction, Ag nanoparticles form the main fraction with the same sizes of about 3–4 nm as a fraction of small inhomogeneities observed in the initial chitosan. Thus, one can conclude that the metal nanoparticles formed and were located in the small pores (3–4 nm of size) of the polymer matrix. They also formed associates with sizes up to 30 nm but not so large as the size of the largest inhomogeneities in the initial matrix.

**Figure 3:**
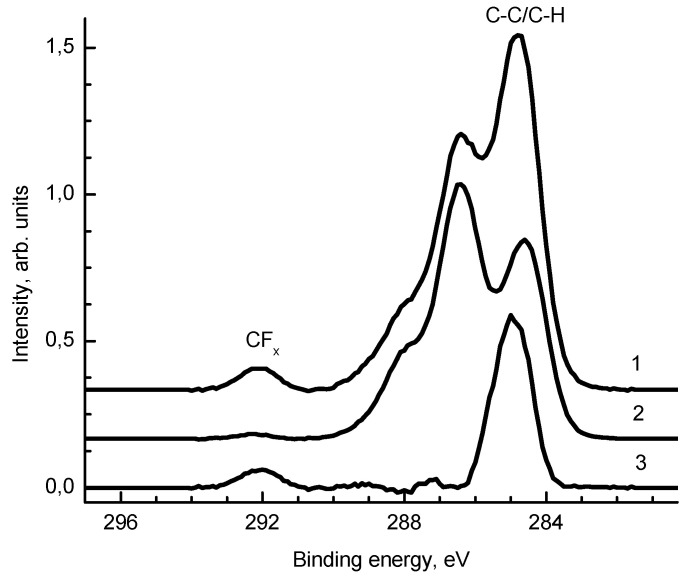
The C 1s spectra of samples 1 and 2 and their difference spectrum 3.

Figure 3 shows the C 1s spectra of samples 1 and 2. After the deposition of silver, an increase in the relative concentrations of the C-C/C-H and CF_x_ groups in the C 1s spectrum was observed. The O 1s spectra of samples 1 and 2 are similar. 

**Figure 4:**
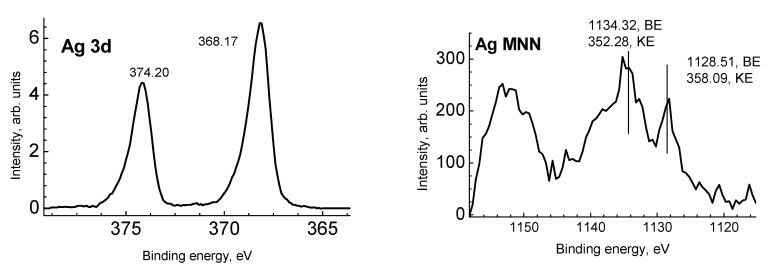
The Ag 3d and Ag MNN spectra of sample 1.

Figure 4 presents the Ag 3d photoelectron and Ag MNN Auger spectra. To identify the chemical state of silver atoms, both the binding energy (BE) and modified Auger parameter were used [32]. A compilation of the literature data for Ag-Ag and Ag-O bonds [32,33,34,35,36,37,38,39,40,41,42,43]. The binding energies of the Ag 3d_5/2_ peak for Ag, Ag_2_O, and AgO according to the NIST XPS database [42] are in the ranges 367.9–368.4, 367.7–368.4, and 367.3–368.1 eV, respectively. Taking into account that the most accurate binding energy of the Ag 3d_5/2_ peak for solid silver is 368.327 eV, the corresponding kinetic energy for the Ag M4N45N45 peak is 357.855 eV, and the Auger parameter is 726.182 eV [41,44], one can conclude that the chemical state of silver in sample 1 corresponds to the Ag0 state.

**Figure 5:**
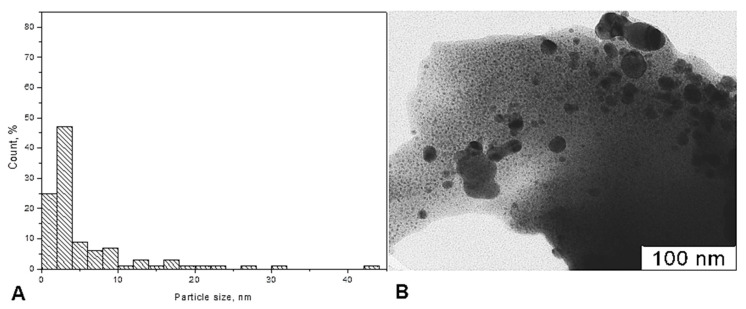
(**A**) Particle size analyser data, (**B**) Transmission electron microscopic image of Ag NPs Chitosan nanocomposites.

The TEM results showed the formation of spherical silver nanoparticles “dark field” with a size ranging from 4 to 10 nm was recorded in the next 92% of the total percentage, homogeneously distributed in the chitosan matrix. The TEM photos do not show aggregate between the Ag NPs. This is mainly due to the carboxylic coordination on the silver surface, which can effectively prevent aggregation and fusion between nanoparticles. The particles are small and there are some large clusters on the chitosan powder surface (Figure 5).

### 3.2. Analysis of Toxins for Patulin and Citrinin

**Figure 6:**
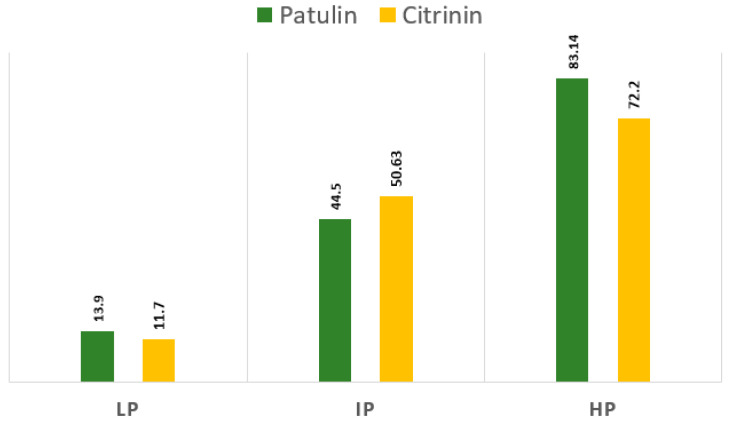
The quantitative results of patulin and citrinin assays of *P. expansum* isolates, including three levels of mycotoxins, high producer (HP), intermediate producer (IP), and low producer (LP).

The quantitative results of the *Penicillium* spp toxicity test are shown in Table 2. In total, 3 of the 17 tested isolates produced both patulin and citrinin toxins. The productive *P. paneum* (isolate 2) produced 91.10 parts per billion (ppb), and *P. expansum* isolate produced 83.14 and 72.20 ppb patulin and citrinin, respectively. *P. citrinum* isolate 3 produced 197.20 ppb of citrinin (Table 3 and Figure 6). Isolates vary in the type and concentration of toxins produced. It is noteworthy that most of the *P. expansum* isolates tested (100%) were bitoxic (co-producers of patulin and citrinin), with very high patulin production. To evaluate the antifungal activity, three different fungal isolates varying significantly in mycotoxin-producing abilities were selected (highly producer (HP), intermediate producers (IP), and low producers (LP)).

### 3.3. Antifungal Activity

**Figure 7:**
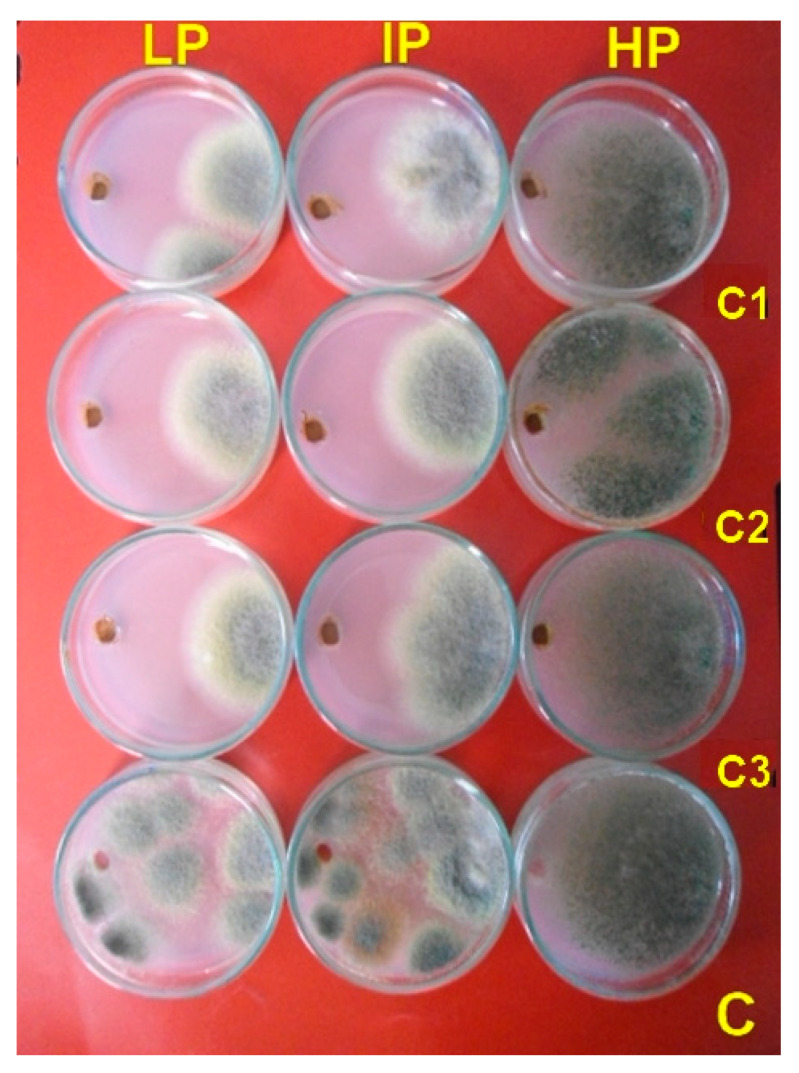
Antifungal activity for different concentration of Ag-Chit-NCs (C1 = 0.30, C2 = 0.60, and C3 = 0.100 mg·mL^−1^) against *P. expansum* isolated from feeds by the plate assay. All Petri dish treatments were incubated at 28 °C for 10 days.

The colloidal solutions of Ag-Chit-NCs at various concentrations (0.30, 0.60, and 0.100 mg·mL^−1^) with particle sizes ranging from 4–10 nm were used to evaluate the inhibitory activity against dairy cattle toxicosis *P. expansum* through treatment duration. *P. expansum* LP and IP isolates were entirely inhibited at all tested concentrations of Ag-Chit-NCs of 0.30 mg·mL^−1^ after 7 days of incubation. The developed nanocomposites showed a low inhibition zone with the efficient producer of high levels of patulin and citrinin (HP isolate). The control treatment did not enhance the antifungal activity (Table 3 and Figure 7). To our knowledge, the present study establishes the first application of Ag-Chit-NCs as an antifungal against *P. expansum* collected from feed samples.

### 3.4. Characterize Nanocomposites-Fungal Protein Interactions

**Figure 8:**
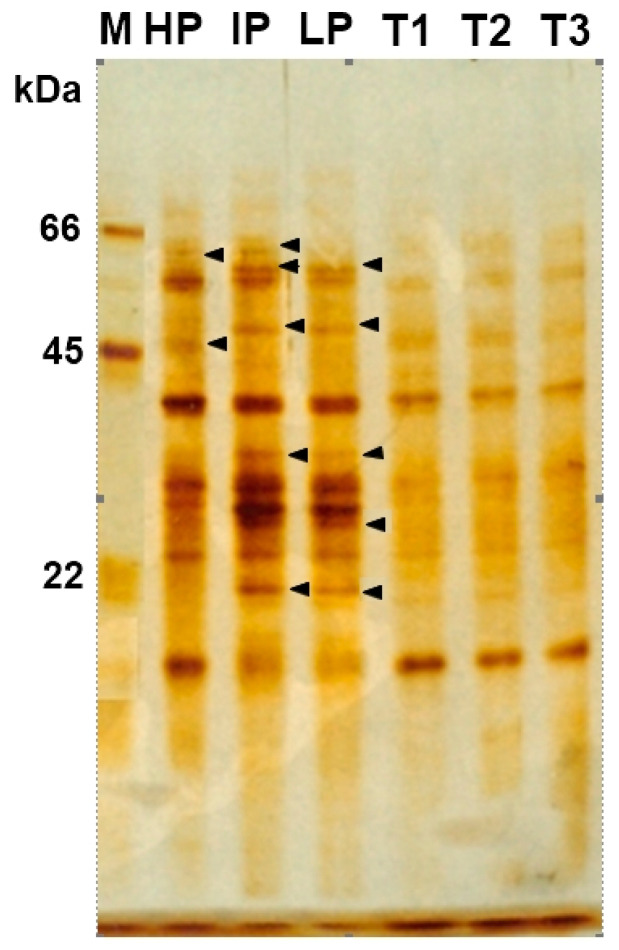
Protein expression profile of silver-stained SDS-PAGE from *P. expansum* mats at a high Ag-Chit-NCs concentration. Lane M contains a protein marker. Protein bands are shown in the black arrow (seven reduced band). The protein standards with molecular weights ranging from 66, 45, and 22 kDa were used. *P. expansum* isolates, including three levels of mycotoxins, high producer (HP), intermediate producer (IP), and low producer (LP). The same isolates treated with Ag-Chit NCs as a fellow T1, T2, and T3, respectively.

SDS-PAGE gel electrophoresis was performed to screen the modification of gene expression of *P. expansum* treated with various concentrations of Ag-Chit-NCs. Many protein bands were found in *P. expansum*, which were exposed and not exposed to Ag-Chit-NCs. The protein profile in the control group shows a clearer protein band. Specifically, there were three major bands in the protein maker, including 66, 45, and 22 kDa, respectively. Seven bands completely disappeared after treatment with 0.90 mg of Ag-Chit NC, with molecular weights of 62, 60, 47, 38, 27, and 20 kDa. *P. expansum* secretes four different proteins, with molecular weights between 60, 47, 38, and 10 kDa (Figure 8). *P. expansum* isolates under pressure with the presence of Ag-Chit-NCs in higher concentrations can reduce the production of some proteins. The amount of protein expression in each treatment varies with the control, exactly where the band intensity of each treatment was changed as compared with their control. Compared to untreated wells, seven bands were degraded in *P. expansum* isolates.

### 3.5. Analysis of the Binding/Degradation of Fungal Genomic DNA

**Figure 9:**
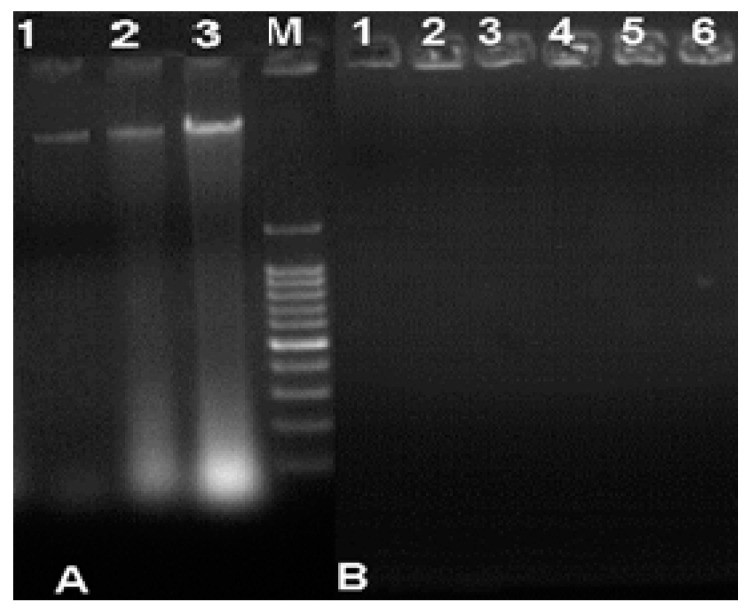
Agarose gel electrophoretic pattern of the fungal genomic DNA treated with 10 μL Ag-Chit-NCs. **A**: Lanes 1–3: DNA for untreated *P. expansum* isolates, Lanes 1: *P. expansum* 1, Lane 2: *P. expansum* 2, Lane 3: *P. expansum* 3. **B**: Lanes 1–6: three *P. expansum* isolates DNA treated with Ag-Chit nanocomposites (30, and 40 μg·mL^−1^) showed total damage to fragmented DNA bands.

Agarose electrophoresis of genomic DNA from fungi exposed to Ag-Chit-NCs showed fragmented and smeared DNA compared to the controls. Control DNA shows the main band of intact/unaffected genomic DNA, where no significant damage occurred. In contrast, fungi treated with different concentrations of silver nanoparticles show induction depending on the termination of DNA strands, which are characterized by increased DNA fragmentation (Figure 9). In this study, the genotoxicity manifested by Ag-Chit-NCs was demonstrated by DNA fragmentation after treatment, especially at concentrations of 30 and 40 μg·mL^−1^ with high concentrations of Ag-Chit-NCs.

### 3.6. Fungal Morphology Observation

**Figure 10:**
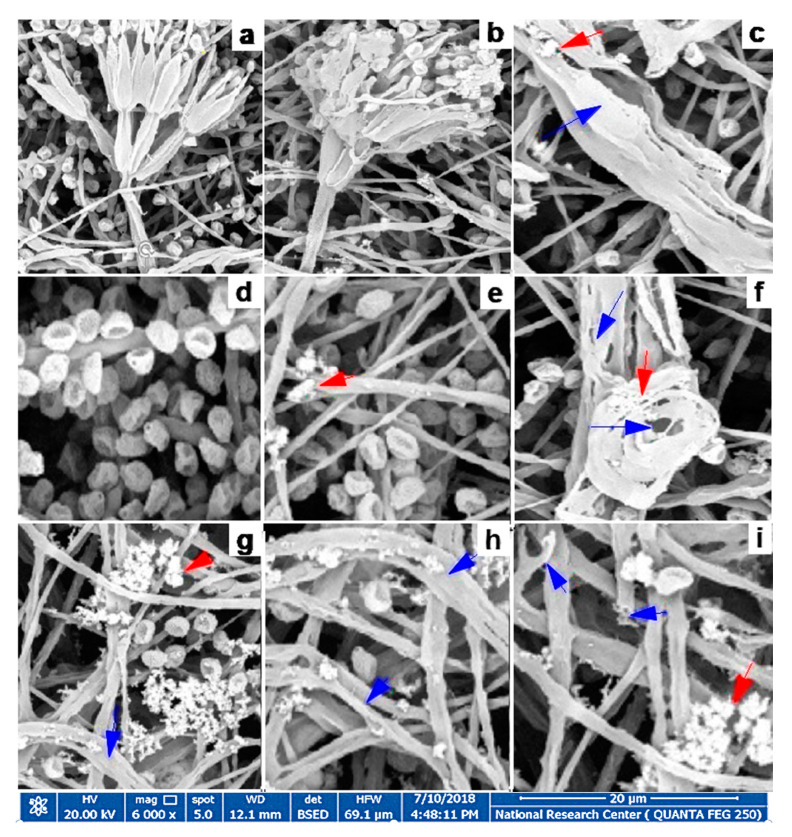
Scanning electron microscope (SEM) photographs of *P. expansum* after treatment with Ag-Chit-NCs (**a**) *P. expansum* have globose smooth-walled and ellipsoidal conidia and are from 3 to 3.5 µm long (**d**). Red arrows refer to Ag-Chit NCs’ upper different fungal structures while blue arrows refer to the morphological changes in the fungal hyphae, such as irregular branching (**b**,**c**), loss of linearity and warty surfaces (**c**,**f**,**h**), collapsed cell, formation of a layer of extruded material (**g**,**h**,**i**), a small vesicle (**i**), SEM were the markedly shriveled, crinkled cell walls, and flattened hyphae of the fungi (**g**–**i**), hyphal cell wall, and vesicle damage (**c**–**i**).

*P. expansum* isolates grown on PDA plates showed the characteristic mycelial and conidiospores morphology, with lengthened, normal, and homogenous hyphae of a constant diameter with smooth external surfaces and rounded apices (Figure 10a,d). Healthy mycelia of the untreated (control) consisted of conidiophores formed of smooth stipes 200 to 500 µm long and ending in typically triverticillate penicilli (presence of one or more branches on the stipe. The length of the metulae ranged from 12 to 15 µm, while that of the tightly packed phialides was 8 to 11 µm, Phialide were flask-shaped and more elongated. The morphological changes in *P. expansum* treated with Ag-Chit-NCs were investigated by HR-SEM, and alterations in conidiophores, metulae, phialides, and mature conidia characteristics were observed. Substantial morphological changes in the cell wall surface of the fungal mats were observed and conidiophore improvement was abnormal, where mycelia and conidiophores were shriveled compared to the untreated controls. The cell walls became thinner, shriveled, crinkled, and showed a decreased cytoplasmic content and modifications of the membrane integrity, and the fungal conidiophore started to shed its spores. Additionally, SEM offered evidence of the morphological changes due to exposure to Ag-Chit NCs, which included irregular branching (Figure 10b,c), loss of linearity and warty surfaces (Figure 10c,f,h), collapsed cell, the formation of a layer of extruded material (Figure 10g,h,i), a little vesicle (Figure 10i), hyphal cell wall, and damaged vesicles (Figure 10c–i).

## 4. Discussion

The present research aimed to evaluate the fungicide effect of Ag-Chit-NCs on *P. expansum*, which potentially has a toxic effect on animal feed. In this study, the qualitative production of patulin and citrinin was examined using HPLC. All tested *P. expansum* isolates were bitoxic and their ability to produce patulin toxin was very high. Of the 51 isolates tested, 33 (65%) produced patulin and only one (2%) citrinin [45]. In a Spanish study, three out of five *P. expansum* strains (60%) produced patulin and five out of eight *P. expansum* strains (63%) produced citrinin [46]. A Slovak study reported the possibility of in vitro patulin production in 37 (82%) of 45 *P. expansum* isolates, and 22 isolates (49%) capable of producing citrinin [47]. Most of the isolates of *P. expansum* (21/23, 91%), when tested for toxigenicity, were bi-toxigenic, producing citrinin and a very high amount of patulin [48]. Four mycotoxins, including patulin (PAT), mycophenolic acid (MPA), cyclopiazonic acid (CPA), and Roquefortin C (ROC), are found in fresh ingredients, contrary to the penicillium toxin formation, which only occurs during storage. Toxins produced from *Penicillium* species are reported in corn silage and are associated with health problems in cattle [2]. The current study provides new information about the potential risks of patulin and citrinin in the silage of corn and other feeds.

However, the precise mechanism of Ag-Chit-NCs through which the reaction takes place is still largely unknown. Numerous reports have investigated the electrostatic attraction between the cell membranes of negatively charged microorganisms, such as bacteria, viruses, and fungi, and positively charged nanoparticles, which is very important for the antibacterial regime of these particles [49]. It has been suggested that silver nanoparticles with large surface positions can easily produce Ag^+^ by binding to the sulfhydral (-SH) practical groups of proteins and consequently denaturing proteins [50,51]. Silver nanoparticles can also cause denaturation and destruction of proton pumps by binding to fungal surface proteins, increasing the membrane permeability, or lipid bilayer proteins, which ultimately leads to the disruption of cell membranes [52,53]. Proteomic analysis in *P. expansum* was assayed by two-dimensional electrophoresis (2-DE) after chitosan treatment. Proteins related to DNA or protein biosynthesis, and carbohydrate metabolism were reduced, while proteins involved in antibiotics resistance and defense response were improved after chitosan treatment, which can explain the antifungal activity of chitosan against *P. expansum* [54].

**Figure 11:**
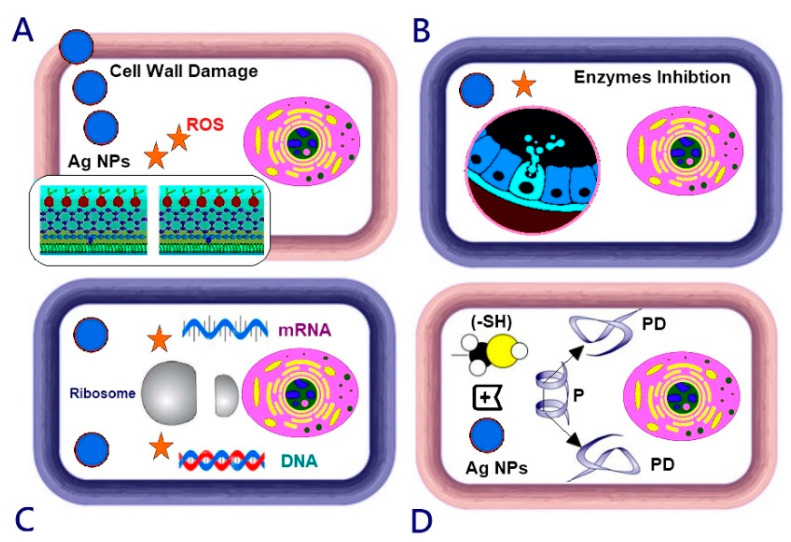
The mechanistic approach of the antifungal action indicating ROS generation induced by Ag-Chit-NCs (star). Degradation and leakage of cell walls and cell membranes, electronic transport chain disturbances, enzyme inhibition, decomposition or destabilization of ribosomes, DNA and mRNA damage, and protein denaturation.

Based on the previous research [55], it is probable that silver nanoparticles can damage the transportation system, causing outflow intracellular ions and silver ions to accumulate and inhibit processes, such as metabolism and respiration. Ag NPs also engage with protein thiol groups, which are important for the ability of microbial respiration [56]. Ag NPs can also interact with phosphorus-containing compounds, such as DNA; interfere with the replication process; or preferably attack the respiratory chain. It has also been speculated that bacterial cells associated with silver nanoparticles can absorb silver ions, which can facilitate the generation of reactive oxygen species and consequently cause necrobiosis [57]. Silver nanoparticles can also cause DNA damage, and damage the ability of replication, cell walls, cell walls, mitochondria, chromatin, and ribosomes [53]. Chitosan can penetrate the fungal cell membrane and bind to its DNA, which can inhibit mRNA synthesis and thereby interfere with the accumulation of proteins and essential enzymes [58]. The fungicide activity of chitosan against some plant pathogenic fungi, such as *P. expansum* and *A. alternata*, may be caused by membrane damage due to interactions between protonated amino groups and negatively charged cell surface proteins [21,59]. Reactive oxygen species (ROS) molecules (hydrogen peroxide, hydroxyl radicals, and peroxide) play a critical role in biological systems, and the multiplied content of intracellular ROS molecules destroys cellular components, such as DNA, RNA, proteins, and lipids, and even damages the integrity of toxic oxygen toxins. The cytotoxic effect of Ag-Chit-NC is the result of active physicochemical interactions of silver atoms with intracellular protein functional groups in addition to nitrogen and phosphate groups in the DNA molecule (Figure 11).

Concerning the effects of chitosan-based nanomaterials on various fungal pathogens, the undoubted interactions between chitosan NP molecules and the polyanionic structure of microbial cell membranes cause destabilization of the cell membranes. This results in the leakage of intracellular content and then the death of the pathogen. Impaired protein synthesis and membrane destabilization are most likely the primary and secondary modes of chitosan antimicrobial activity [60]. Additionally, the mechanism of nano-chitosan includes the penetration of low-molecular-weight chitosan into the cellular DNA binding site, which further inhibits the synthesis of RNA and protein, as shown by Mostafa et al. [61]. It is believed that the current type of nanocomposite motion may be far more complicated than assumed above; such studies must clarify the proper mechanism.

Silver nanoparticles are completely deformed hyphal mats and lysed into small and elastic fragments. The remaining mycelium appears as collapsed particles that are fused with the internal components of the cell that are released. The results obtained are consistent with those found in other studies [62]. Nano-chitosan causes fungal mat accumulation, and structural changes, such as excessive branching, cell wall swelling, and reduced hyphal size, which have been observed in *P. expansum* and *Rhizopus stolonifera* [63].

## 5. Conclusions

The current study provides new information linked to the potential risks of patulin and citrinin in corn silage and confirms that mycotoxins and *P. expansum* can pose risks to human and animal health. The obtained results highlight the importance of being able to control the occurrence of *P. expansum* in dairy cattle feed. These results indicate that silver nanoparticles/chitosan nanocomposites can be further investigated as effective fungicides for applications in agriculture and food safety. Ag-Chit-NCs are a feasible and effective supplement for reducing mycotoxins in animal feed. The use of developed nanocomposites can be recommended, e.g., because disinfectant spray can be a successful chance to prevent food and feed contamination caused by mycotoxin-producing fungi. Ag-Chit-NCs are an active biopesticide that represent a new level of inexpensive biological control system that can be used in crops. There is a lack of clear conclusions about the chitosan-based NCs mode of action, cell membrane damage, DNA transformation, ROS production, or other mechanisms. Therefore, more studies are needed to find the right mechanism. More large-scale studies are needed. These results support the consideration of the possibility of mycotoxins occurring in dairy cows, such as patulin and citrinin. We recommend routine monitoring of animal feed colonization by other toxigenic fungi before and during harvest and identification of relevant mycotoxins. The use of silver nanoparticles/chitosan nanocomposites must also be monitored closely for side effects and examined on a case-by-case basis, especially for agricultural commodities.

## Figures and Tables

**Table 1:** Elemental composition of the samples determined from the survey spectra.

Sample	C	O	Ag	Si	Zn	N	Cl	F
1	66.38	24.38	1.25	4.06	0.22	3.70	-	-
2	59.43	27.72	-	3.71	0.51	4.93	0.14	3.56

Sample 1: chitosan (HMWC), Sample 2: chitosan matrix.

**Table 2:** *Penicillium* spp. isolates used in mycotoxins assay.

Isolate Code	*Pencillium* Species	Feed Source	Mycotoxin Concentration (ppb)	
			Patulin	Citrinin
1.	*Penicillium paneum*	maize silages	37.40	ND
2.	*Penicillium paneum*	maize silages	91.10	ND
3.	*Penicillium paneum*	maize silages	80.36	ND
4.	*Penicillium expansum*	cottonseed meals	0.00	40.19
5.	*Penicillium expansum*	maize silages	13.90	11.70
6.	*Penicillium expansum*	maize silages	44.50	50.63
7.	Penicillium expansum *	maize silages	83.14	72.20
8.	*Penicillium citrinum*	maize-Bran	ND	120.0
9.	*Penicillium citrinum*	dry natural forage	ND	88.56
10.	*Penicillium citrinum*	dry natural forage	ND	197.20
11.	*Penicillium citrinum*	sunflower oilcake	ND	77.13
12.	*Penicillium citrinum*	maize silages	ND	0.0
13.	*Penicillium citrinum*	dry natural forage	ND	60.22
14.	*Penicillium griseofulvum*	dry natural forage	67.50	ND
15.	*Penicillium verrucosum*	maize silages	ND	97.18
16.	*Penicillium verrucosum*	maize silages	ND	143.0
17.	*Penicillium verrucosum*	maize silages	ND	24.00

ND, not detected, * highly producer isolate.

**Table 3:** Zone of inhibition of Ag-Chit-NCs against *Penicillium expansum* isolates, including three levels of mycotoxins, high producer (HP), intermediate producer (IP), and low producer (LP) collected from maize silage.

Concentrations (mg·mL^−1^)	*Penicillium expansum* Strains			
	LP	IP	HP	Mean
0.30	3.16	3.48	2.50	3.16
0.60	3.66	3.56	2.45	3.66
0.90	3.79	3.82	2.39	3.79
Control	0.00	0.00	0.00	0.00
LSD (*p* < 0.05)	0.363	0.234	0.220	-

Inhibition zone (cm) and each value is mean of five replications (three plates).
